# Bibliographical

**Published:** 1872-06

**Authors:** 


### BIBLIOGRAPHICAL.

A Text-Book of Practical Medicine, with Particular Reference to Physiology and Pathological Anatomy. By Dr. Felix von Niemeyer, Professor of Pathology and Therapeutics, Director of the Medical Clinic of the University of Tubingen. Translated from the eighth German edition by special permission of the author, by George H. Humphreys, M. D., and Charles E. Hockley, M. D. Vols. 2. New York: D. Appleton & Co., 549 and 551 Broadway; 1872.

Medical readers will be pleased to learn that the Appletons have just issued, as above, a new and revised edition of Niemeyer's great work. The scope and design of the volumes before us, the author states to be two-fold: first, to give a picture of disease which shall be life-like and faithful to nature as possible, instead of being a mere theoretical scheme; second, so to utilize the more recent advances of pathological anatomy, physiology, and physiological chemistry, as to furnish a clearer insight into the various processes of disease. Niemeyer's work is one of the recent advances made in the field of practical medicine, and no physician can afford to deprive himself of it. The late edition now presented to the American physician contains two new articles on "Relapsing Fever" and "Suette Melaise," and some extensive remarks on the employment of bromide of potassium in the treatment of epilepsy, hypodermic injections of corrosive sublimate in syphilis, and the employment of the stomach tube in dilitation of the stomach, etc.

The translators have done their work well, while the typographical appearance is that of the usual excellence of the Appletons.

A Treatise on Diseases of the Nervous System. By William A. Hammond, M. D., Professor of Diseases of the Mind and Nervous System, and of Clinical Medicine in the Bellevue Hospital Medical College, etc., etc. With forty-eight illustrations. Second edition, revised and corrected. New York: D. Appleton & Co.; 1872.

The second edition of this valuable work has, within three months after the publication of a large edition, been called for by the demand for it. It has undergone thorough revision, and is in every way improved. Professor Hammond gives in his work facts drawn from his own large observation and experience, and brings them, with his educated judgment, to bear upon the diseases discussed. The volume "embraces an introductory chapter, which relates to the instruments and apparatus employed in diagnosis and treatment of diseases of the spinal cord; and five sections of these; the first treats of disease of the brain; the second, diseases of the spinal cord; the third, cerebro-spinal diseases; the fourth, diseases of nerve cells; and fifth, diseases of the peripheral nerves. The great practical importance of the work is such as should place it in the hands of every physician in the land.

CLINICAL LECTURES ON THE DISEASES OF WOMEN. By SIR JAMES Y. SIMPSON, Bart., M. D., D. C. L., late Professor of Midwifery in the University of Edinburgh. Edited by ALEX. R. SIMPSON, M. D., Professor of Medicine and Midwifery and the Diseases of Women and Children in the University of Edinburgh. New York: D. Appleton & Co.; 1872.

This valuable volume contains much of the labor of its distinguished author. It will commend itself to the scientific physician of every land. For the first time ten additional lectures are given the profession in the work, and the entire volume, comprising fifty lectures, forms one of the most attractive and valuable works on the diseases of women to be found in any language. It is an indispensable work for reference, and no medical library can be complete without it.

LECTURES ON AURAL CATARRH; OR THE COMMONEST FORMS OF DEAFNESS AND THEIR CURE. By PETER ALLEN, M. D., Fellow of the Royal College of Surgeons, Edinburgh, etc., etc. New York: William Wood & Co., 27 Great Jones Street; 1872.

The title of this work will at once attract the attention of the practical physician. While the author does not pretend to produce an exhaustive treatise upon aural disease, yet the subjects embraced in it cover a wide field. The subjects are systematically arranged and rendered useful and practical. The author recognizes the important fact that "the busy practitioner" has no time to search and sift among endless records of cases, ranged under multitudinous classes, to obtain any assured clue for his guidance." He has therefore "taken considerable pains in the construction of what may be called the ground plan" in the series of lectures presented; and expresses the hope "that they will be found by an earnest reader cf some genuine help in telling him how to manage, what to look for, and where to find the disease, in a case of aural catarrh." We most heartily commend the work to our readers.

THE AMATEUR MICROSCOPIST; OR VIEWS OF THE MICROSCOPIC WORLD. A hand book of microscopic manipulation, and microscopic objects. By JOHN BROCKLESBY, A. M., Professor of Mathematics and Natural Philosophy in Trinity College, Hartford. Illustrated in 247 figures on wood and stone. New York: William Wood & Co.; 1871.

This is one of the most beautiful and instructive volumes on microscopy ever issued. The illustrations are full of interest and handsomely executed, and the wonders of the microscopic world are brought to view with distinctness and beauty. The volume is so attractive it will surely find a place in every library.

THE TREATMENT OF VENEREAL DISEASES. A Monograph on the Method pursued in the Vienna Hospital, under the direction of Professor VON SIGMUND; including all the Formulæ. By M. H. HENRY, M. D., Surgeon to the New York Dispensary—Department of Venereal and Skin Diseases, etc., ect. Adapted and arranged from the German. New York: William Wood & Co.; 1872.

The author is one of the most thorough venereal surgeons in the country, and his monograph will be highly prized by every earnest student and practitioner. The formulæ alone will give the volume a large circulation, embracing, as they do, all in use in the Vienna Hospital, the value of which, with the remarks of the author, cannot be too highly esteemed by the busy practitioner.

SELECTIONS FROM FAVORITE PRESCRIPTIONS OF LIVING AMERICAN PRACTITIONERS. By HORACE GREEN, M. D., LL. D., President of the Faculty and Emeritus Professor of the Theory and Practice of Medicine in the New York Medical College, etc., etc. New York: John Wiley & Son, 15 Astor Place; 1872.

This book of formulæ gives at one glance the favorite prescriptions of the best and ablest medical men of the country. They were collected in the following manner: "For many years the author's rooms for the treatment of patients have been visited daily by medical men from all parts of the Union, who have called on him, either through curiosity or a desire to observe every improvement in practical medicine." He thinks one thousand physicians have thus annually called upon him. Desiring to secure information from every section, he "accustomed himself for many years, when visited by experienced, practical physicians, to request of them copies of some of their favorite prescriptions." From these, thus gathered, with those of his own, the work is compiled, which cannot fail to meet the favor of practical physicians everywhere, as giving a condensed view of the practice of the best living physicians of the country.

A NUMBER of books from the above houses and others, not included, remain on our table awaiting notice, which want of space in this issue precludes. They will be attended to in our next.

